# Measuring empathy in youth and young adults with mild intellectual disabilities or borderline intelligence in residential care - The validation of an adapted basic empathy scale: The BES-MID

**DOI:** 10.1016/j.heliyon.2024.e31050

**Published:** 2024-05-15

**Authors:** Evelyn Heynen, Anniek Borghuis, Ron Pat-El, Xavier Moonen, Geert Jan Stams

**Affiliations:** a Open University, Heerlen, the Netherlands; b Universiteit van Amsterdam, Amsterdam, the Netherlands

**Keywords:** Empathy, Mild intellectual disability, Empathy scale, Youths

## Abstract

Youths with mild intellectual disability (MID) are assumed to show impaired cognitive and affective empathy. However, the use of linguistic and conceptual complex empathy measures set limits to the valid and reliable assessment of empathy in youth with MID or borderline intelligence (BI). The objective of the present study was to evaluate the validity and reliability of the adapted BES-MID as an instrument for assessing cognitive and affective empathy in youth and young adults (12–24years) with MID/BI. The current study was conducted in a sample of N = 146 youth and young adults with MID or BI Results from Confirmatory Factor Analyses confirm the two-factor structure of affective and cognitive empathy for the BES-MID. While the original BES cannot be used to assess empathy in youth and young adults with MID or borderline intelligence, the BES-MID showed satisfactory validity and reliability in youth and young adults with MID or borderline intelligence. Despite some limitations and the need for further research, the current study has resulted in a valid and reliable empathy scale (BES-MID) for assessing cognitive and empathic abilities in youth and young adults with MID or borderline intelligence, which is important for future empathy research in youth and young adults with MID or borderline intelligence.

## 1 Introduction

One of the main goals of human development is to become a person who can empathize with others, and is able to ‘understand and share another's emotional state or context’ [1] Empathy is considered to be the evolutionary mechanism behind altruism, prosocial behaviour, human civilization, and subsequently desistance from violence [2], and it is strongly associated with prosocial behaviour and moral judgement, even from a cross-cultural perspective [4], although, the translation of empathy into prosocial behaviour remains complex [6].

Previous research indicates that having a Mild Intellectual Disability or borderline intelligence (MID/BI, IQ = 50–85) is associated with a lack of empathy [7], inadequate reactions to the emotion ‘sadness’ [8], biased perception of someone's emotions [9], and distorted empathic skills [10,11], which all may increase the risk for the development of antisocial behaviour [12]. Persons with MID/BI show difficulties in abstract thinking and perspective taking, tend to take abstract information too literally (and act accordingly), and have difficulties in distinguishing jokes from serious information [13]. These difficulties often result in social problems, and for a small group can set the stage for criminal behaviour if multiple risk factors accumulate [14]. On the other hand, Kasari and colleagues (2003) found that children with MID/BI showed affective reactions in response to a person in distress, and were able to empathize with certain emotions such as happiness, anger, and fear.

Individuals with MID/BI show a limited working memory capacity, reduced verbal understanding skills, show behavioural problems, and sometimes fail to comply with the rules and expectations of the society they live in due to a lack of sufficient skills to adequately adapt their behaviour to the expectations of others [[15], [16], [17], [18]].

Research indicates that methods to measure emotion recognition in people with MID/BI are unfit, and the deviations of emotion recognition within this population are merely a reflection of their perceived environment, indicating that deficits in emotion recognition are limited to IQ-related information processing [9]. Research on empathy measures which can objectively measure concrete components of empathy is sparse [19]. It is plausible that previous studies that looked into empathic abilities in people with MID/BI used methods that do not fit for this group of people. For instance, it is known that individuals with MID/BI have problems with understanding negatively phrased items, long and complex sentences, abstract formulations, and that they have a limited vocabulary [20,21]. Furthermore, research has shown that people with MID/BI have problems answering questions with many options [20]. Results from the systematic review of Cabedo Peris, (2020) showed that the use of the Basic Empathy Scale (BES; 7) is increasing, although the research areas in which it is being implemented are currently being broadened. This questionnaire has been translated into many different languages, including Dutch [22], German [23], Spanish [24] and Polish [25]. A recent review indicated that the BES is appropriate to be used in general population groups [26]. Previous research indicates that many items of the BES were not reliable in research among juvenile offenders (e.g., 22; 23; 24), which may partly be explained by the high prevalence of individuals with MID/BI in populations of offenders ([27]; in the Netherlands up to 70 %, NJI, 2017; [[16], [28], [29]]). Prevalence rates of offenders with MID/BI are higher in youth as compared to adults, and also higher in cases of BI as compared to cases of MID [16]. These findings support the assumption that due to difficulties in the comprehension of complex textual and conceptual information in people with MID/BI the BES may be an invalid and unreliable measure of empathy for this group. Previous research has shown that adapting the language of a questionnaire to the level of verbal functioning in people with MID/BI can result in less reading mistakes and a better understanding of textual information [30,31]. The present study aims to assess if the adapted BES-MID shows an improved model fit for youth and young adults with MID/BI compared to the original BES, and so can be considered a valid and reliable instrument to assess cognitive and affective empathy in youth and young adults with MID/BI. We expect the BES-MID to be a valid and reliable instrument to investigate cognitive and affective empathy in juveniles with MID/BI. We furthermore expect that there is a need for adaptation of the original version of the BES to investigate empathy in youth with MID/BI properly.

## 2 Method

### 2.1 Procedure

The present research employed a correlational, cross-sectional survey design. To recruit participants with MID/BI, we approached care organizations that serve adolescents and young adults with MID/BI, providing them with information about the study. Upon receiving permission from the organization, clients who met the inclusion criteria were invited to participate, a process facilitated by either the researcher or an organization employee. Those willing to participate scheduled appointments for questionnaire completion. If an individual was under 16, parental or guardian consent was required. Participants aged 16 and older signed an informed consent, which was ID adapted and explained further by the researcher as needed. Caregivers were not present during questionnaire completion to prevent potential influence on participant responses. The original BES or the BES-MID was randomly assigned for completion, with questions read aloud by the researcher if reading was challenging for the participant. After finishing the questionnaire, participants received a chocolate bar as reward. Ethical approval has been obtained by Ethical Review Committee Psychology and Neuroscience Maastricht University on 25.04.2017 reference number: 178_06_04_2017.

### 2.2 Participants

Participants were 146 youth and young adults (*n*_*female*_ = 59/40.41 %; *n*_*male*_ = 87/59.59 %) age 12–24 years (*mean* = 16.49, *SD* = 2.64) with Mild Intellectual Disability or Borderline Intelligence (MID/BI) who received receive outpatient or residential treatment based on their previous diagnosis of MID/BI. The population of interest were adolescents with MID/BI highly at risk for juvenile offending. The present study did not have access to the specific IQ data of the participants, and no IQ measurements were taken. All included youth did have an IQ range between 50 and 85, which is in line with the definition of MID as having an IQ score between 50 and 70 [32]. In the Netherlands individuals with a borderline intelligence (BI) score (IQ score between 71 and 85) who suffer from deficits in adaptive functioning and additional serious problems are entitled to receive treatment that fits individuals with MID/BI if this helps them to recover [33], and thus people with IQ-scores between 50 and 85 are designated as people with MID/BI in the Netherlands. That is why both youth with MID/BI and youth with borderline intellectual functioning were included in the present sample (MID/BI). All participants had the Dutch nationality and voluntarily participated in the study based on informed consent. Participants were randomly assigned to complete the BES or the BES-MID. Finally, a total of 65 participants completed the original BES, and 81 the BES-MID.

### 2.3 Basic empathy scale (BES)

The original BES is a 20-item questionnaire based on a two-factor model of affective and cognitive empathy. Affective empathy is measured by eleven items and cognitive empathy by nine items. An example question of the cognitive empathy factor is: ‘*I understand my friend's happiness when she/he does well at something*’. An example of the affective empathy factor is: ‘*After being with a friend who is sad about something, I usually feel sad*’. The questions are answered on a 5-point Likert scale, ranging from 1 = totally not applicable to 5 = totally applicable. In this study, the Dutch version of the BES translated by Van Langen et al. (2015) was used. This Dutch version of the BES has been found to be sufficiently reliable for both factors (cognitive empathy, *α* = 0.72, and affective empathy, *α* = 0.81) and was found to adequately fit the two-factor model [22].

### 2.4 Adaptation of the basic empathy scale for MID (BES-MID)

Items of the Dutch Basic Empathy Scale [22] were rephrased based on the rules for the Dutch ‘language for all’ (Taal voor allemaal, [34]). Rules for applying ‘language for all’ include avoiding abstract language, using active words and using short sentences. A first draft of the adapted version was checked and commented by two youth with MID/BI, resulting in the final version of the BES-MID, which also consisted of twenty items divided in two subscales: cognitive (9 items) and affective (11 items) empathy. Items were divided into three categories, namely: items about friends and family, items about strangers and items about the self. As negatively phrased items are expected to be too difficult to understand for individuals with MID/BI, the number of negatively phrased items was reduced to four. Two of these items contain a denial, which is formatted in bold and underlined manner i.e. “*I do* ***not*** *find it important how my friends are feeling*”. Answers are given on a 3-point Likert scale ranging from answering “no” [1], “a little” [2] to “yes” [3], which are respectively colored red, orange and green. An example of the BES-MID questionnaire can be requested from the first author of the study.

### 2.5 Statistical analysis

We conducted analysis in R version 4.2.2 [35]. Several packages were utilized for data manipulation, visualization, and modeling. SPSS data files were loaded using the ‘haven’ package [36]. Missing data were imputed using the ‘mice’ package [37]. Multiple imputation was performed using the predictive mean matching (PMM) method with five permutations to account for missing data. PMM is a widely used and validated method for handling missing data in statistical analyses [38]. For each missing value, PMM finds a set of similar cases in the dataset based on a set of predictor variables and then draws an imputed value from the distribution of observed values for that set of cases. This approach preserves the relationships between variables in the dataset while reducing bias and increasing the precision of estimates [38]. To evaluate the BES and the BES-MID the ‘lavaan’ package [59] was used to conduct confirmatory factor analysis (CFA). In all analyses, the two-factor model, with 11 items measuring affective empathy and 9 items measuring cognitive empathy, was imposed to the data. The ‘MVN’ package was used to test multivariate normality using the Mardia test [39]. The Mardia test indicated severe skewness in the BES (z-skewness = 1798.62, p < 0.001) and severe skewness and kurtosis in the BES-MID (z-skewness = 2219.67, p < 0.001; z-kurtosis = 6.31, p < 0.001). Due to the ordinal measurement level of the items and the violation of the assumption of multivariate normality, the CFA was conducted using the pairwise maximum likelihood (PML) estimator. PML is particularly advantageous when dealing with non-normally distributed data or data that violate the assumption of multivariate normality. Unlike some other robust estimators, such as Diagonally Weighted Least Squares (DWLS), which assumes normality, PML does not rely on such stringent assumptions, making it more robust in scenarios where such assumptions are violated. Additionally, PML tends to provide more precise parameter estimates, especially when dealing with small sample sizes [40].

Two CFAs were conducted using the ‘cfa’ function from the ‘lavaan’ package. The first CFA was conducted on the BES dataset, and the second CFA was conducted on the MID dataset. Given that this study makes use of a small dataset (*N* < 15) to interpret a model's fit, the following indicators were used based on for sample-size accounting fit criteria [41]: CFI and TLI scores above 0.95, and TLI scores above indicate good fit [42] whereas CFI and TLI scores above 0.90 indicate acceptable fit [43]. Root mean squared error of approximation (RMSEA) below 0.06 and standardized root mean residual SRMR below 0.12 indicates good fit, while RMSEA below 0.08 indicate acceptable fit. Factor loadings ideally should be > 0.70, but at the least have to be ≥ 0.30. Several items in the BES and BES-mid were reverse coded. To account for negative wording effects in the model, the residual variances of these items were correlated [[43], [44], [45], [46]]. Calculations of Cronbach's alpha were conducted in SPSS 24. We reported a Cronbach's Alfa of 0.6–0.8 to be acceptable and alpha >0.8 to be good.

## 3 Results

### 3.1 Data screening

Prior to analysis, scores for all items were examined for accuracy of data entry, distributions, univariate outliers. No items had extreme scores (IQR >3). Five missing values in total were found in five items of the BES, and six missing values in total were found in 5 items of the BES-MID. These were imputed with predictive mean matching based on all other items in the questionnaire in order to preserve power.

### 3.2 confirmatory factor analysis

Characteristics of the participants are presented in Table 1. Both CFA models demonstrated statistically significant chi-square values, indicating a lack of perfect fit between the hypothesized model and the observed data. However, given the sensitivity of the chi-square statistic to sample size, additional fit indices were considered (see Table 2).

**Table 1:** Demographic characteristics of respondents.

Condition			*n*	*Min-max*_*age*_	*Mean*_*age*_	*SD*_*age*_
MID	BES	female	28	13–23	16.57	2.15
		male	37	12–24	16.62	2.60
	BES-MID	female	31	13–23	16.48	2.70
		male	50	12–24	16.34	2.92

**Table 2:** Fit Statistics for the CFA models for the full BES and BES-MID.

Model	**χ**^**2**^	df	p	CFI	TLI	RMSEA	SRMR
**BES**	217.946	154	<0.001	0.745	0.686	0.080	0.144
**BES-MID**	223.033	163	<0.001	0.896	0.879	0.067	0.167

For the BES model, only the RMSEA indicates an acceptable fit, whereas all other fit measures fall below or above their thresholds suggesting a less than optimal fit to the data. The BES-MID model demonstrated an acceptable fit across several measures. The CFI and TLI values were very close to the threshold of 0.90, indicating acceptable fit to the data. Additionally, the RMSEA value met the criteria for acceptable fit. The SRMR however fell far from the threshold of good fit. To enhance model fit, items with standardized estimates below 0.30 were removed. This refinement was applied to both the BES and BES-MID. For the BES-MID, this resulted in the removal of items 5 (cognitive) and 20 (affective). Regarding the BES, a considerable number of items exhibited standardized estimates close to zero, leading to the removal of the BES items 12 (cognitive), 2, 7, 8, 13, 17, and 18 (affective). It's noteworthy that the removal of these items, except for BES19 and BES20, eradicated all negatively worded items from the BES. The results of the Confirmatory Factor Analysis (CFA) on the revised models are presented in Table 3. Retaining only those items with standardized estimates ≥0.30 in the initial model yielded good fit for the BES across all fit indices, with close-to-good fit for the SRMR (0.126). The removal of two items in the BES-MID resulted in acceptable fit for some fit indices. Comparative Fit Index (CFI) (0.907) and Root Mean Square Error of Approximation (RMSEA) (0.07) demonstrated acceptable fit, while the Tucker-Lewis Index (TLI) was very close to acceptable fit (0.892). However, the SRMR did not indicate acceptable fit (0.169).

**Table 3:** Fit Statistics for the CFA models for the shortened BES and BES-MID.

Model	χ^2^	df	p	CFI	TLI	RMSEA	SRMR
**BES**	71.675	63.000	<0.001	0.939	0.925	0.046	0.126
**BES-MID**	183.423	131.000	<0.001	0.907	0.892	0.070	0.169

For the BES-MID the model that best fitted the data consisted of cognitive empathy (8 items) and affective empathy (10 items), whereas item 5 of the original cognitive empathy scale and item 20 of the affective empathy scale had to be removed to obtain satisfactory model fit. See appendix A for all factor loadings.

For the BES, Cronbach's alpha was insufficient for the cognitive subscale *α* = 0.54 and acceptable for the affective subscale *α* = 0.64. For the BES-MID Cronbach's alpha was satisfactory for both the cognitive subscale *α* = 0.74 and affective subscale, *α* = 0.74 (see Table 3).

## 4 Discussion

The present study contributes to the limited body of research on empathy in youth and young adults with MID or borderline intelligence. Results of the present study suggest that in order to be a valid and reliable measure to use in youth and young adults with MID or borderline intelligence there is a need for adaptation of the original version of the BES to assess cognitive and affective empathy. Results indicate that the construct validity of the original BES is insufficient to use with youth and young adults with MID/BI. Model fit and reliability of the adapted BES-MID proved to be satisfactory, indicating that this instrument is a valid and reliable measure of cognitive and affective empathy in youth and young adults with MID/BI.

Results showed that item 5 (“*I find it hard to see if my friends are scared*”), which represents a cognitive emotional state, had to be removed to obtain a good model fit. An explanation might be that youth tend to mask their real attitudes when dealing with strong and negative emotions ([23,47,48]). This item refers to strong and negative emotions, such as sadness, fright, and anxiety, and was also removed in the validation study of the Dutch BES in a mixed sample of youth and young adults in the general population and delinquent youth in detention [22], and in the validation study of the German BES in a sample of delinquent youth and young adults in youth detention [23].

Also item 20 (“*I get scared when I watch a horror movie*”), which is part of the affective subscale, had to be removed to obtain a good model fit. A possible explanation is that this item can be perceived as unrealistic compared to the real-world violence, especially by individuals with MID/BI, because they often have to deal with disadvantaged environments ([23,24,47,48]). Both removed items focus on negative emotions and on feeling afraid. There is evidence that people with MID/BI overall have a limited range of coping strategies, and the types of strategies that they use to regulate their emotions are not always effective [49], resulting in self-regulation difficulties, which refer to the ability to regulate emotion, behaviour, and cognition [51]. Research indicates that children and adolescents with MID/BI have fewer self-regulation abilities than their typically developing peers ([52,53]), and that they also have difficulties regarding emotion regulation, cognition, behaviour, and physiology in response to stressful events or circumstances [49], which might point towards specific difficulties in dealing with emotions and prosocial behaviour ([5,50,54]). Notably, at least three reviews showed that lack of empathy and/or prosocial behavior in individuals with ASD can be treated effectively, including those with mild and moderate intellectual disability ([[60], [61], [62]]). Finally, there is support from research that adolescents with MID/BI experience lower levels of angry mood in their daily life compared to their peers without intellectual disabilities [54].

The present study has some limitations. Although self-reports of psychological difficulties are considered reliable for youth and young adults with MID/BI (e.g., [55]), they possibly experience more difficulties in reporting about internal processes, such as dealing with emotions, rather than about concrete behaviours [20]. Youth and young adults with MID/BI may therefore be more inclined to give socially desirable answers and acquiescence [56,57]. However, there is no well-validated instrument available yet to assess socially desirable responding and acquiescence in individuals with MID/BI. We therefore could not assess divergent validity of the BES-MID by examining the association between empathy and social desirability as well as acquiescence. Another limitation is about current assessment of mild intellectual disability or borderline intelligence. No assessment of adaptive behaviour was done, which is a distinctive element of the classification MID/BI in the DSM-5 [32]. Furthermore, respondents’ IQ scores varied from 50 to 85 and participants were considered to belong to one group (MID/BI), while it is possible that research at a subgroup level would have influenced results [20]. In future validation research of the BES-MID both intelligence and adaptive behaviour should be assessed in the recruitment process of MID/BI respondents. Finally, in future research, including a larger sample, convergent and divergent validity of the scale should be assessed, by examining its correlations with empathy related constructs (e.g. *Test of Emotional perception,* [58]), such as personality and prosocial behaviour, also from a cross-cultural perspective [4,7].

Despite these limitations, the current study is the first to assess empathy with the BES-MID in youth and young adults with MID/BI, using an instrument that is adapted to the cognitive and linguistic abilities of youth and young adults with MID/BI. Results of our study provide empirical evidence that the BES-MID is a valid and reliable empathy measure for affective and cognitive empathy in adolescents and young adults with MID/BI, which is an important step towards better understanding their often complex social behaviour.

## 5 Data availability statement

The datasets generated during and/or analysed during the current study are available from the corresponding author on reasonable request.

No funding was received for conducting this study. The authors have no competing interests to declare that are relevant to the content of this article.

Ethical approval has been obtained by Ethical Review Committee Psychology and Neuroscience Maastricht University on 25.04.2017 reference number: 178_06_04_2017.

## CRediT authorship contribution statement

**Evelyn Heynen:** Writing – review & editing, Writing – original draft, Supervision, Methodology, Investigation, Formal analysis, Conceptualization. **Anniek Borghuis:** Writing – original draft, Formal analysis. **Ron Pat-El:** Writing – original draft, Software, Methodology, Formal analysis, Data curation. **Xavier Moonen:** Writing – original draft, Supervision. **Geert Jan Stams:** Writing – review & editing, Supervision.

## Declaration of competing interest

The authors declare that they have no known competing financial interests or personal relationships that could have appeared to influence the work reported in this paper.

## Figures and Tables

**Table:** Appendix AStandardized Regression weights.

**BES**				
Item number	Scale/Item	Scale/Item Dutch		
	cognitive empathy	cognitieve empathie	Standardized Estimates	
			MODEL 1	MODEL 2
BES 3	I can understand my friend's happiness when she/he does well at something.	Ik begrijp dat mijn vrienden blij zijn als ze iets goed hebben gedaan	0.519	0.505
BES 6	I find it hard to know when my friends are frightened	Ik vind het moeilijk om in te schatten wanneer mijn vrienden bang zijn	−0.491	−0.498
BES 9	When someone is feeling ‘down’ I can usually understand how they feel	Wanneer iemand zich rot/‘down’ voelt, begrijp ik hoe diegene zich voelt	0.485	0.457
BES 10	I can usually work out when my friends are scared.	Ik kan zien wanneer mijn vrienden bang zijn	0.543	0.568
BES 12	I can often understand how people are feeling even before they tell me	Ik begrijp hoe mensen zich voelen, vaak al voordat zij dit mij vertellen	0.090	–
BES 14	I can usually work out when people are cheerful	Ik zie meestal wel wanneer mensen blij zijn	0.574	0.557
BES 16	I can usually realise quickly when a friend is angry	Ik heb het snel door als een vriend/vriendin kwaad is	0.399	0.391
BES 19	I am not usually aware of my friend's feelings	Ik heb het vaak niet door hoe mijn vrienden zich voelen	−0.350	−0.366
BES 20	I have trouble figuring out when my friends are happy.	Ik vind het moeilijk om te snappen wanneer mijn vrienden blij zijn	−0.479	−0.482
	affective empathy	affectieve empathie		
BES 1	My friend's emotions don't affect me much.	De gevoelens van mijn vrienden doen mij niet zoveel	0.340	0.293
BES 2	After being with a friend who is sad about something, I usually feel sad.	Nadat ik bij een vriend ben geweest die ergens verdrietig over is, voel ik me meestal ook verdrietig	−0.211	–
BES 4	I get frightened when I watch characters in a good scary movie	Ik word bang wanneer ik naar een griezelfilm kijk	−0.379	−0.369
BES 5	I get caught up in other people's feelings easily.	Ik laat me makkelijk meeslepen in de gevoelens van anderen	−0.496	−0.506
BES 7	I don't become sad when I see other people crying.	Ik wordt niet verdrietig als ik andere mensen zie huilen	−0.014	–
BES 8	Other people's feelings don't bother me at all	De gevoelens van andere mensen doen me helemaal niets	−0.045	–
BES 11	I often become sad when watching sad things on TV or in films.	Ik wordt verdrietig wanneer ik zielige (nare) dingen zie op TV of in films	−0.823	−0.735
BES 13	Seeing a person who has been angered has no effect on my feelings.	Als ik iemand zie die boos is, doet me dat niets	0.010	–
BES 15	I tend to feel scared when I am with friends who are afraid.	Als ik met vrienden ben die bang zijn, voel ik me ook bang	−0.736	−0.799
BES 17	I often get swept up in my friend's feelings.	Ik ga mee in de gevoelens van mijn vrienden	−0.215	–
BES 18	My friend's unhappiness doesn't make me feel anything.	Als mijn vrienden ongelukkig zijn, doet dit mij niets	0.187	–
**BES-ID**				
Item Number	Scale/Item	Scale/Item Dutch		
	cognitive empathy	cognitieve empathie	Standardized Estimates	
			MODEL 1	MODEL 2
BES-MID3	I understand that my friends are happy when they do something well	Ik snap dat mijn vrienden blij zijn als ze iets goed doen.	0.545	0.541
BES-MID4	I can see how my friends are feeling	Ik zie aan mijn vrienden hoe zij zich voelen.	0.646	0.648
BES-MID5	I find it hard to see if my friends are scared	Ik vind het lastig om te zien of mijn vrienden bang zijn.	−0.203	–
BES-MID6	I find it hard to understand when my friends are happy	Als mijn vrienden blij zijn, vind ik dat moeilijk om te snappen.	−0.402	−0.408
BES-MID8	I notice when my friends are angry	Ik merk als mijn vrienden kwaad zijn	0.595	0.590
BES-MID11	I see when my friends are scared	Ik zie wanneer mijn vrienden bang zijn.	0.745	0.725
BES-MID15	When someone feels bad, I understand why	Als iemand zich rot voelt, begrijp ik waarom.	0.730	0.719
BES-MID16	I can see how people are feeling	Ik zie aan mensen hoe ze zich voelen.	0.617	0.617
BES-MID18	When someone is happy, I can see	Als iemand blij is, zie ik dat.	0.696	0.696
	affective empathy	affectieve empathie		
BES-MID1	I do **not** find it important how my friends are feeling	Ik vind het **niet** belangrijk hoe mijn vrienden zich voelen.	0.382	0.388
BES-MID2	When a friend is sad, I am also sad	Als een vriend(in) verdrietig is, ben ik ook verdrietig.	−0.573	−0.562
BES-MID7	When my friends are scared, I am also scared	Als mijn vrienden bang zijn, ben ik ook bang.	−0.310	−0.294
BES-MID9	When my friends have a feeling I also get that feeling	Als mijn vrienden een gevoel hebben dan krijg ik dat gevoel ook.	−0.415	−0.406
BES-MID10	When my friends are unhappy, I feel bad for them	Als mijn vrienden ongelukkig zijn, vind ik dat erg.	−0.542	−0.546
BES-MID12	What other people feel is **not** important to me	Wat andere mensen voelen, vind ik **niet** belangrijk.	0.344	0.347
BES-MID13	I easily get caught up in another person's feelings	Ik ga makkelijk mee in wat iemand anders voelt	−0.765	−0.771
BES-MID14	I get sad when I see someone else cry	Ik word verdrietig als ik iemand anders zie huilen.	−0.723	−0.723
BES-MID17	When I see that someone is angry, I feel bad for that person	Als ik zie dat iemand boos is, vind ik dat vervelend voor die persoon.	−0.633	−0.635
BES-MID19	I get sad when I see something sad on TV	Ik word verdrietig als ik iets zieligs zie op TV.	−0.622	−0.608
BES-MID20	I get scared when I watch a scary movie	Ik word bang als ik naar een horror-film kijk.	−0.111	–
